# Correlation analysis between biomechanical characteristics of taekwondo double roundhouse kick and effective scoring of electronic body protector

**DOI:** 10.3389/fphys.2023.1269345

**Published:** 2024-01-11

**Authors:** Mengyao Jia, Lin Liu, Ruifeng Huang, Yong Ma, Shijie Lin, Qian Peng, Jun Xiong, Zhaoyi Wang, Weitao Zheng

**Affiliations:** ^1^ Engineering Research Center of Sports Health Intelligent Equipment of Hubei Province, Wuhan Sports University, Wuhan, China; ^2^ Research Center of Sports Equipment Engineering Technology of Hubei Province, Wuhan Sports University, Wuhan, China; ^3^ Key Laboratory of Sports Engineering of General Administration of Sports of China, Wuhan Sports University, Wuhan, China; ^4^ Department of Physical Education, Northwest Polytechnical University, Xi’an, China; ^5^ School of Competitive Sports, Wuhan Sports University, Wuhan, China

**Keywords:** taekwondo, protector and scoring system, double roundhouse kick, biomechanics, effective scoring

## Abstract

**Objective:** To explore the inherent relationship between lower limb biomechanical indicators and effective scoring values of double roundhouse kick (DRK) by taekwondo athletes, and to find key biomechanical factors that trigger effective scoring.

**Methods:** Using the DAEDO Protector and Scoring System (PSS) in conjunction with the Vicon optical motion capture system and Kistler 3D force plate, kinematic and dynamic indicators of the front kicking motion were obtained from 12 professional taekwondo athletes (18.00 ± 2.20 years, 182.15 ± 8.62 cm and 70.00 ± 14.82 kg). The correlation between kinematics, dynamics, and scoring values was initially analyzed using bivariate linear correlation. Subsequently, based on the results of the linear correlation analysis, a stepwise regression analysis was performed to establish a stepwise regression equation.

**Results:** The results reveal that during the First Hit, there is a significant positive correlation (r > 0, *p* < 0.05) between peak hip flexion angular velocity of the dominant leg, knee abduction angle, and peak foot horizontal plane linear velocity of the non-dominant leg with effective score. On the other hand, peak ankle flexion angular velocity of the non-dominant leg, peak foot sagittal plane linear velocity, peak hip abduction angle, and peak hip flexion angle of the dominant leg exhibit a significant negative correlation (r < 0, *p* < 0.05) with effective score. These correlations hold statistical significance (DW> 1.023). During the Second Hit, there is a significant positive correlation (r > 0, *p* < 0.05) between peak ankle internal rotation angular velocity of the dominant leg, foot coronal plane linear velocity, hip adduction angular velocity, and peak ankle internal rotation moment of the non-dominant leg with effective score. Conversely, peak hip flexion angle of the dominant leg shows a significant negative correlation (r < 0, *p* < 0.05) with effective score. All these variables have a statistically significant impact on effective score (DW > 1.023).

**Conclusion:** Explosive power, body posture, adequate terminal velocity, and body rotation have an association with effective scoring of the electronic protector. The peak angular velocity of the ankle joint of the dominant leg and the peak linear velocity of the foot horizontal plane of the non-dominant leg significantly contribute to the effectiveness score of the electronic protector.

## Introduction

The combat sport of athletic taekwondo is a kind of competition that emphasises direct physical interaction and high levels of confrontation (Pieter et al., 2012). It features rapid technical speed, brief periods of contact, and a wide variety of different strategies (Huang et al., 2009). When referees manually judge taekwondo matches (Gao et al., 2010), they can only decide on a subjective level if the technical moves are standardized and if the strikes are effective. This is because there are blind spots in the referee’s field of vision caused by the overlap of the athletes’ mutual contact (Lu et al., 2010; Zhang et al., 2014). In 2010 Asian Games, World Taekwondo (WT) began incorporating cutting-edge technology into the sport (Woo et al., 2013; Ko et al., 2014; Moenig, 2015), and the rules of taekwondo competition were rewritten extensively to usher in the era of a DAEDO PSS. WT has officially recognised a DAEDO PSS, therefore it is extensively utilised in important tournaments like the Olympics and the World Championships (Fang, 2011; Zhang and Guan, 2017).

The PSS used in competitions as a whole is relatively expensive, and its accompanying software is subject to regular updates. Some teams may have invested less in the PSS and most players have to practise in the conventional gear. When it comes to training competitions, many technical actions can be scored with traditional protective gear, but the sensors of the PSS cannot be judged as effective, leaving coaches and athletes frustrated. As a result, they are eager to learn the parameters of the striking actions that can cause the PSS to score, so that they can better prepare their athletes for competition (Gasparutto et al., 2021).

Focusing on striking the opponent’s torso or head with forceful lower limb strikes is essential in competitive taekwondo (Geßlein et al., 2020), and whether the PSS determines effective scoring is related to whether the force value of the strikes exceeds the set threshold and whether it hits the valid part (Razman and Chong, 2019). Striking power is proportional to striking velocity, and to achieve sufficient striking velocity, the athlete must rapidly mobilise the muscles to generate strength in a coordinated manner, causing the foot to stomp hard in order to obtain the ground reaction force, which in turn causes a spatial change in the limb, allowing the athlete to complete the technical movement. Technical maneuvers in Taekwondo are separated into punches and kicks (Jin and Liu, 2012), with kicks being the predominant technique (Fu and Qian, 2010). The DRK is the sole technique that hits twice, is a frequent technique in international competition (Huang and Meng, 2009), and is one of the most important scoring methods for elite athletes (Shen and Gao, 2018). From 2015 to 2016, the scoring of all kicking techniques to the torso raised from one point to two. Changes in competition regulations have resulted in a shift in Taekwondo battle patterns toward stronger body motions and more intense conflict. Significant increases in kinematic markers, heart rate, energy expenditure, and overall physiological load suggest that the new regulations require athletes to have a higher level of physical fitness (Tornello et al., 2014; Janowski et al., 2021).

Technical analysis is an analytical tool commonly used by coaches and sport scientists to better understand specific motor skills and provide a basis for improving skill performance. Researchers use models such as deterministic modelling to identify factors and biomechanical properties associated with performance so that coaches can make appropriate changes to improve an athlete’s skill level based on the evidence. Such models have been successfully applied to a number of sports, particularly ice-snow sports and athletics, and these models have identified key technical characteristics of athletic performance in a number of sports. Although technical analysis is frequently used in taekwondo related research, there is very limited information on systematic technical analysis in taekwondo.

The majority of the literature on the DRK action today concerns the technical and tactical shifts necessitated by the new rules (He and Pang, 2019; Lin and Gao, 2020), the biomechanical characteristics of individual techniques (Aloui et al., 2022; Liu et al., 2023), and the impact of sports training on the kick itself (Sant'Ana et al., 2021; Ojeda-Aravena et al., 2021); however, the relationship between effective scoring values and the kinematics and kinetics of the kick itself, assuming the PSS and triggering scoring, has received surprisingly little attention. For this reason, we use a DAEDO PSS to mimic the conditions of a real competition and determine whether or not there is a correlation between the biomechanical characteristics of the participants’ lower limbs (peak joint angle, peak joint angular velocity, peak foot linear velocity, peak joint moment, peak value of vertical ground reaction force) and the PSS’s effective scoring, and explore the variables that contribute significantly to effective scoring with DAEDO PSS. Therefore, we hypothesised that, there was a significant effect between the angular velocities of ankle and knee flexion and extension of the striking leg, as well as the internal and external rotation moments of the pivot leg ankle, the vertical ground reaction force and the effective score values at the moment of both strikes. The scientific judgement of how the DRK can effectively trigger the PSS is made possible, and theoretical support is provided for improving the training effect of the DRK in taekwondo, as well as the effective scoring, by analyzing the internal connection between the effective scoring value and the biomechanical characteristics of the kick.

## Methods

### Participants

Predicted sample size was determined using G*Power 3.1.9.2 (Kang, 2021). The effect size was set at 0.6. At *α* = 0.05 and statistical effectiveness, there had to be at least 12 subjects. Twelve male athletes (all subjects are athletes of level 2 or above) from the Wuhan Sports University Taekwondo sports training team (18.00 ± 2.20 years, 182.15 ± 8.62 cm and 70.00 ± 14.82 kg) were selected for this research and the physical state of the individuals had no effect on their usual movement performance. All participants trained for more than three consecutive years (the average training history is 3.8 ± 2.0 years), 5 days a week, 3 hours per day and have reported no injuries in the past 6 months. They had not engaged in any high-intensity training in the preceding 24 hours.

### Experimental set-up

The motion capture data was collected using 9 infrared high-speed cameras (T40, VICON, United Kingdom, sampling at 200 Hz) and marker balls with a diameter of 14 mm. In order to get force plate data, used KISTLER 3D force plates (9260AA6, Switzerland, sampling frequency 1 kHz, 4 independent force plates 60 * 40 CM). The WT-approved DAEDO PSS (GEN2, PSS-TK STRIKE; Daedo International, Spain), which was used in the 2012, 2016 and 2020 Olympic Games, includes electronic body protectors, electronic socks, signal transmitters, and TrueScore Wireless System software. The dummy was positioned upright, and an PSS was attached to it before the experiment began. The dummy was then linked to the computer through a wireless signal receiver.

### Data collection

The Wuhan Sports University Ethics Committee gave its consent to this experiment (review number: 2022048). At Wuhan Sports University’s Key Laboratory of Sports Engineering for the General Administration of Sports in China, we gathered the information used here. Pre-test preparation: Before beginning, it was necessary to calibrate the VICON 3D motion capture system, KISTLER 3D force plates, and a DAEDO PSS to guarantee that the data from both systems would be consistent. Next, the subjects were asked about their health and asked to sign an informed consent form. They were also told how the experiment would work and what precautions to take. After a 15 min warmup session, subjects were permitted to paste the reflective markers, and after that was done, they were given permission to swiftly strike the dummy with the PSS in accordance with the specified full-field coordinate direction and the location of the target dummy, as well as to change the dummy’s height to correspond with the standard height of the electronic guard at the subject’s striking. As soon as the athlete is ready, the lab tech will verify the PSS signal, adjust the electronic scoring system to the subject’s competitive kilogramme level, and begin collecting data.

Padding and sliding are common ways for taekwondo competitors to create space between themselves and their opponents so that they may finish a move more swiftly and ultimately earn more points. After each action, the same coach determined whether or not the subject’s performance met the technical requirements for a DRK, and Each subject performed five kicks, and then picked three of the best performances to serve as the test sample.

**FIGURE 1:**
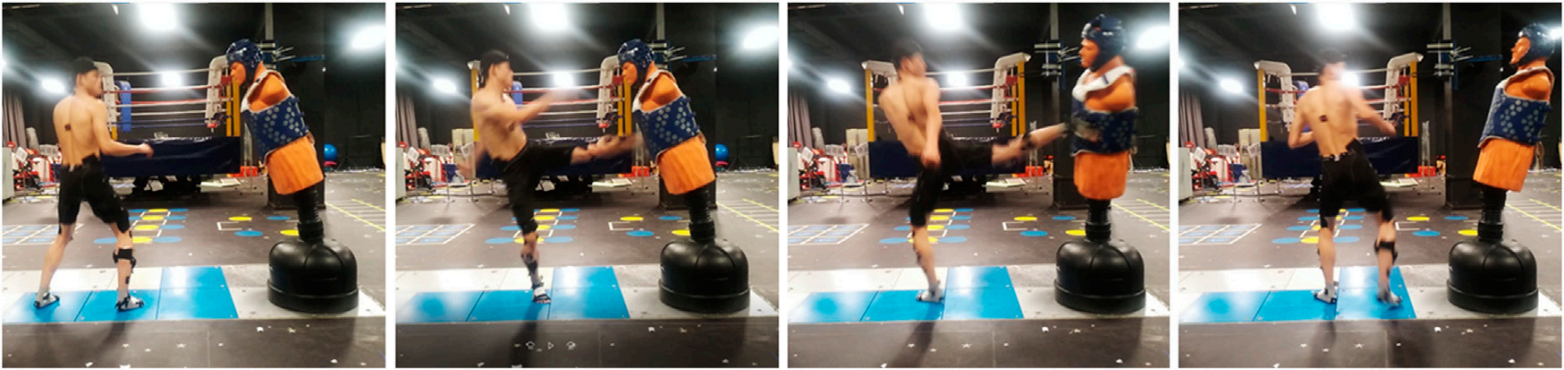
Sequential images of DRK Action. Preparation moment (E1): the instant when the foot of the non-dominant leg leaves the ground and the plate’s ground reaction force is zero. Moment of the first strike (E2): the moment of maximum extension of the non-dominant leg’s knee joint and the moment of minimum angle of the non-dominant leg’s knee joint. Moment of the second strike (E3): maximum extension of the knee of the dominant leg and minimal angle of the knee of the dominant leg. Recovery moment (E4): when the dominant leg’s foot reaches the ground, the ground reaction force of the force plate emerges.

Due to differences in training intensity and lower limb function, taekwondo athletes form a dominant side and a non-dominant side in their lower limbs. The dominant leg is called the dominant leg, preferred leg, or lead leg. In this study, twelve subjects had their dominant leg as the right leg (Figure 1). Meanwhile, we define the striking leg as the leg on the side that strikes the dummy, and the supporting leg as the leg on the same side that makes contact with the ground and provides stability for the body.

**FIGURE 2:**
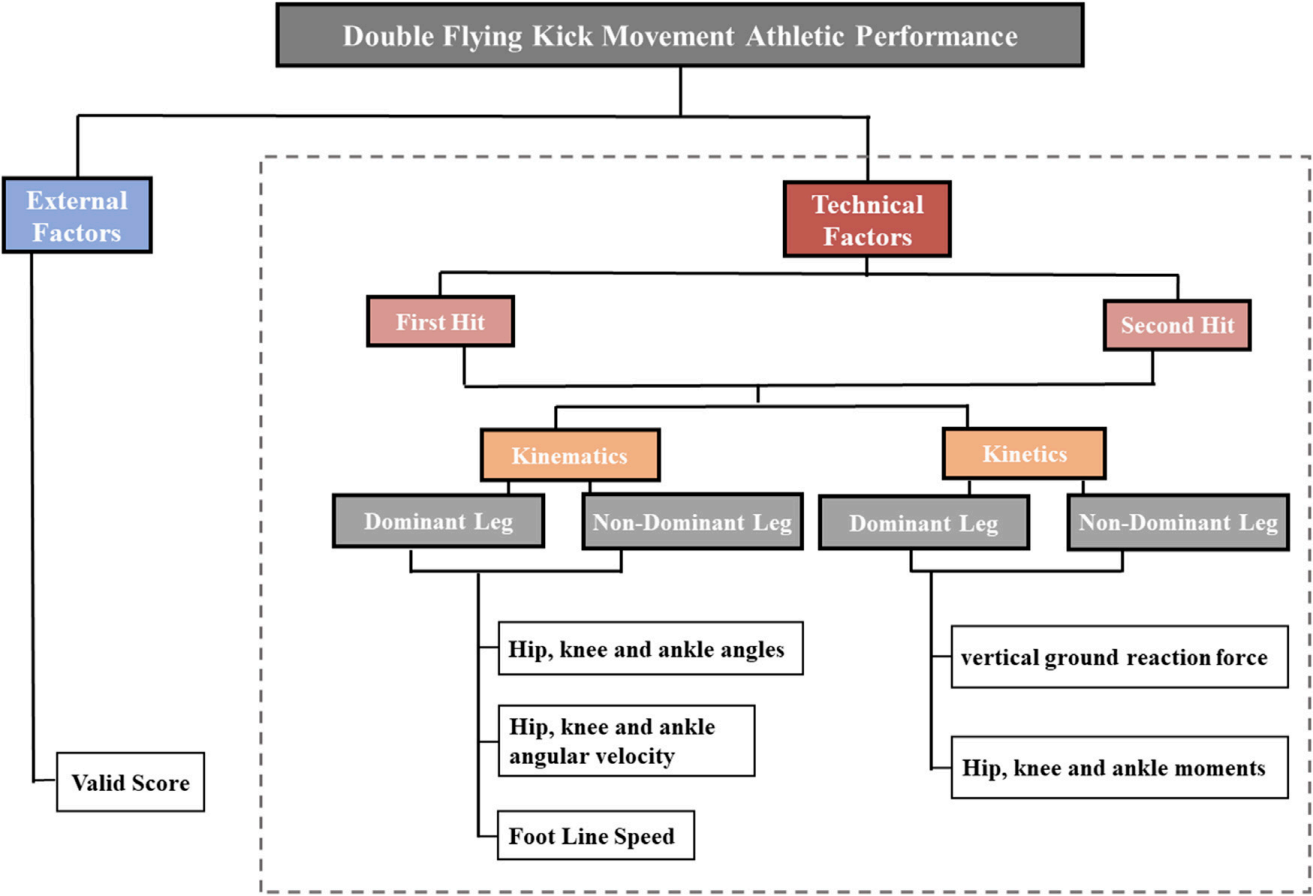
A deterministic model of double Roundhouse Kick. The technical factors are highlighted in the hatched box.

When executing a kicking action, the joint movements of the kicking leg start with rotating the pelvis, followed by simultaneous flexion of the knee joint, adduction/abduction of the hip joint, and rotation of the torso, until contact is made with the target (Ma et al., 2017). Based on the characteristics of the DRK movement, relevant literature (Su et al., 2020; Xu et al., 2020; Sant’ Ana et al., 2017; Wight et al., 2022; Kipp, 2022; Ervilha et al., 2020) and the results of interviews with coaches and athletes, the following striking moment indicators were selected (Figure 2):

1) Kinematics: The dominant and non-dominant leg Peak hip, knee and ankle angles (°), Peak hip, knee and ankle angular velocity (rad/s), foot linear velocity (m/s).
2) Kinetics: The Peak hip, knee and ankle moments (N/kg), peak vertical ground reaction force (N/kg∙m).
3) Effective scoring: The effective scoring value (Score) corresponding to the effective striking action is selected (unitless).

We have identified 104 key factors (refer to Table 1) that may trigger the success or failure of athletes’ scoring, as shown in Table 1. These variables include peak hip, knee, ankle joint range of motion, peak movement speed, peak torque, and peak vertical ground reaction force. All variables were calculated using Visual 3D (C-motion Inc., Germantown, Maryland, United States).

**TABLE 1:** 104 technical factors that can potentially lead to success or failure in triggering scores.

Course of action	Dominant/non-dominant leg	Variant
First Hit	Non-Dominant leg	Peak ankle dorsiflexion angle of the non-dominant leg
		Peak ankle inversion angle of the non-dominant leg
		Peak ankle internal rotation angle of the non-dominant leg
		Peak ankle flexion angular velocity of the non-dominant leg
		Peak ankle inversion angular velocity of the non-dominant leg
		Peak ankle internal rotation angular velocity of the non-dominant leg
		Peak linear velocity in the coronal plane of the foot of the non-dominant leg
		Peak linear velocity in the sagittal plane of the non-dominant leg and foot
		Peak linear velocity in the horizontal plane of the non-dominant leg and foot
		Peak hip flexion angle of the non-dominant leg
		Peak hip abduction angle of the non-dominant leg
		Peak hip internal rotation angle of the non-dominant leg
		Peak hip extension angular velocity of the non-dominant leg
		Peak hip eversion angular velocity of the non-dominant leg
		Peak hip internal rotation angular velocity of the non-dominant leg
		Peak knee extension angle of the non-dominant leg
		Peak knee adduction angle of the non-dominant leg
		Peak knee internal rotation of the non-dominant leg
		Peak knee flexion angular velocity of the non-dominant leg
		Peak knee adduction angular velocity of the non-dominant leg
		Peak knee internal rotation angular velocity of the non-dominant leg
	Dominant leg	Peak ankle dorsiflexion angle of the dominant leg
		Peak ankle pronation angle of the dominant leg
		Peak ankle external rotation of the dominant leg
		Peak ankle flexion angular velocity of the dominant leg
		Peak ankle eversion angular velocity of the dominant leg
		Peak ankle internal rotation velocity of the dominant leg
		Peak foot coronal plane linear velocity of the dominant leg
		Peak foot sagittal plane linear velocity of the dominant leg
		Peak foot horizontal plane linear velocity of the dominant leg
		Peak hip flexion angle of the dominant leg
		Peak hip adduction angle of the dominant leg
		Peak hip internal rotation of the dominant leg
		Peak hip flexion angular velocity of the dominant leg
		Peak hip adduction angular velocity of the dominant leg
		Peak hip internal rotation angular velocity of the dominant leg
		Peak knee extension angle of the dominant leg
		Peak knee abduction angle of the dominant leg
		Peak knee internal rotation angle of the dominant leg
		Peak knee flexion angular velocity of the dominant leg
		Peak knee adduction angular velocity of the dominant leg
		Peak knee external rotation angular velocity of the dominant leg
		Peak vertical ground reaction force
		Peak ankle extension moment of the dominant leg
		Peak ankle inversion moment of the dominant leg
		Peak ankle internal rotation moment of the dominant leg
		Peak hip extension moment of the dominant leg
		Peak hip adduction moment of the dominant leg
		Peak hip external rotation moment of the dominant leg
		Peak knee flexion moment of the dominant leg
		Peak knee adduction moment of the dominant leg
		Peak knee external rotation moment of the dominant leg
Second Hit	Non-Dominant leg	Peak ankle flexion angle of the non-dominant leg
		Peak ankle eversion angle of the non-dominant leg
		Peak ankle external rotation angle of non-dominant leg
		Peak ankle extension angular velocity of the non-dominant leg
		Peak eversion angular velocity of the non-dominant leg
		Peak internal rotation angular velocity of the non-dominant leg
		Peak foot coronal plane linear velocity of non-dominant leg
		Peak foot sagittal plane velocity of the non-dominant leg
		Peak foot horizontal plane velocity of the non-dominant leg
		Peak hip flexion angle of non-dominant leg
		Peak hip abduction angle of non-dominant leg
		Peak hip internal rotation angle of non-dominant leg
		Peak hip flexion angular velocity of the non-dominant leg
		Peak hip abduction angular velocity of the non-dominant leg
		Peak hip internal rotation angular velocity of the non-dominant leg
		Peak knee extension angle of non-dominant leg
		Peak knee abduction angle of the non-dominant leg
		Peak knee internal rotation angle of non-dominant leg
		Peak knee flexion angular velocity of the non-dominant leg
		Peak knee abduction angular velocity of the non-dominant leg
		Peak knee internal rotation angular velocity of the non-dominant leg
		Peak vertical ground reaction force on non-dominant leg
		Peak ankle flexion moment of the non-dominant leg
		Peak ankle inversion moment of non-dominant leg
		Peak ankle internal rotation moment of non-dominant leg
		Peak hip extension moment of the non-dominant leg
		Peak hip adduction moment of the non-dominant leg
		Peak external rotation moment of the non-dominant leg
		Peak knee flexion moment of the non-dominant leg
		Peak knee adduction moment of non-dominant leg
		Peak knee external rotation moment of non-dominant leg
	Dominant leg	Peak ankle flexion angle of the dominant leg
		Peak ankle valgus angle of the dominant leg
		Peak ankle external rotation angle of the dominant leg
		Peak ankle flexion angular velocity of the dominant leg
		Peak ankle eversion angular velocity of the dominant leg
		Peak ankle external rotation angular velocity of the dominant leg
		Peak linear velocity in the coronal plane of the foot of the dominant leg
		Peak linear velocity in the sagittal plane of the foot of the dominant leg
		Peak linear velocity in the horizontal plane of the foot of the dominant leg
		Peak hip flexion angle of the dominant leg
		Peak hip abduction angle of the dominant leg
		Peak hip internal rotation angle of the dominant leg
		Peak hip extension angular velocity of the dominant leg
		Peak hip adduction angular velocity of the dominant leg
		Peak hip external rotation angular velocity of the dominant leg
		Peak knee extension angle of the dominant leg
		Peak knee adduction angle of the dominant leg
		Peak knee internal rotation angle of the dominant leg
		Peak knee flexion angular velocity of the dominant leg
		Peak knee adduction angular velocity of the dominant leg
		Peak knee internal rotation angular velocity of the dominant leg

**FIGURE 3:**
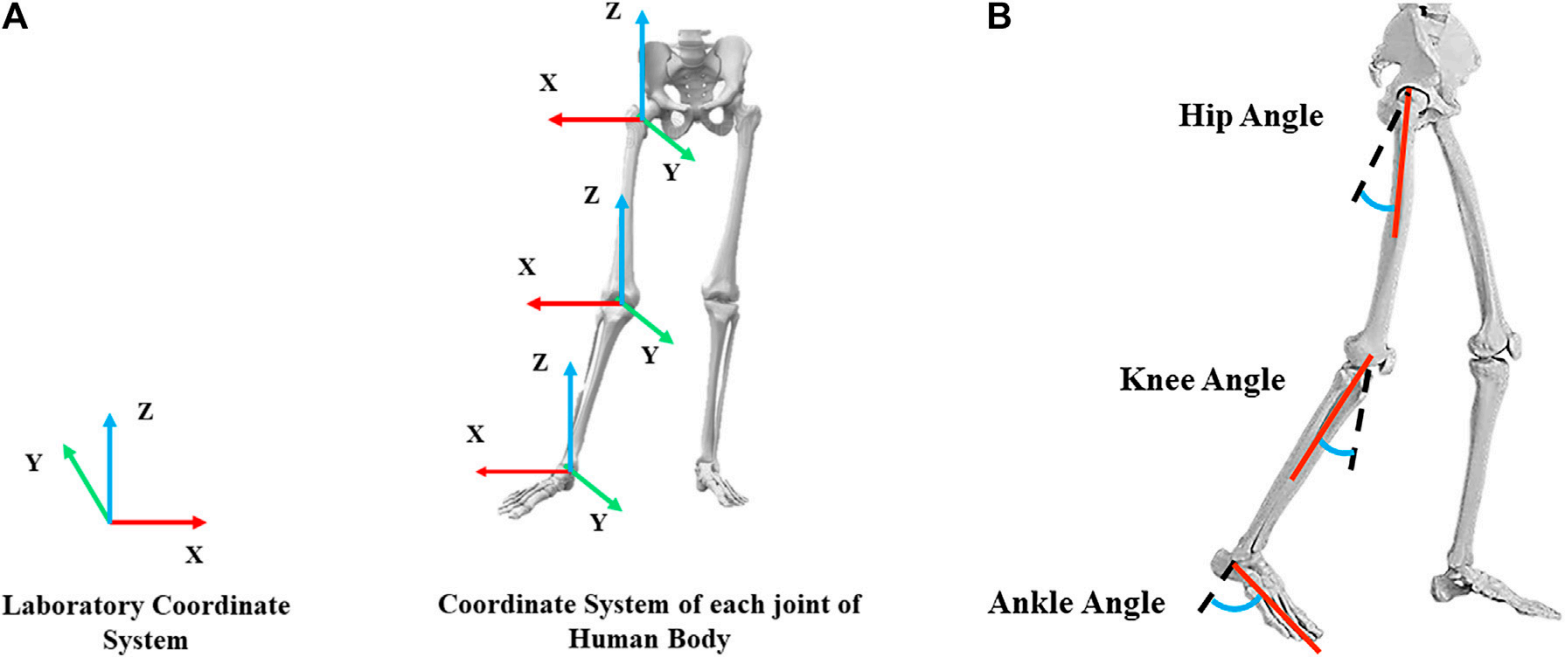
**(A)** Lab coordinates and human joint coordinate system, **(B)** Diagram of hip, knee and ankle joint angles.

The definition of joint coordinate system and joint angle was according to the setting of coordinate system and joint angle in VISUAL3D. Joint coordinate system: coronal axis (*X*-axis, flexion/extension), sagittal axis (*Y*-axis, abduction/adduction), vertical axis (*Z*-axis, rotation inward/outward), as shown in Figure 3A. Joint angles: ankle joint angle: the angle between the foot and calf extension line, knee joint angle: the angle between calf and thigh extension line, hip joint angle: the angle between thigh and trunk extension line, as shown in Figure 3B.

### Data processing

The test data of each subject were exported to C3D files on VICON software after concatenation and complementary points and imported into VISUAL3D software, and static data were used to build model of the lower limbs and calculate the relevant study indicators. The filter cut-off frequencies for kinematic and kinetic data were 10 Hz and 25 Hz, respectively (Sun et al., 2022; Li et al., 2021). The kinetic data were standardized using weight (kg) × height (m) because of the differences in height and weight between individuals.

### Statistical analyses

Statistical analysis of the experimental data was performed using IBM SPSS software (Version 22.0, IBM Corporation, Armonk, New York, United States). The significance level was set at *p* < 0.05. Firstly, the Shapiro-Wilk test was used to analyze the normal distribution of the data. Then, we conducted a linear bivariate Pearson correlation test to analyze the correlation between lower limb kinematics, kinetics, and effective scores in the double flying kick movement. Furthermore, we used stepwise multiple regression analysis to examine the impact of lower limb joint biomechanical parameters on effective scores in the double flying kick movement, and adjusted R2 was used to evaluate the degree of variability explained by the biomechanical parameters. A significance level of *p* < 0.05 indicates statistically significant differences. A statistically significant difference was defined as *p* < 0.05. * indicates *p* < 0.05, ** indicates *p* < 0.01.

## Results

### The correlation between lower extremity kinematic and kinetic characteristics and effective scoring

The correlation results of lower limb kinematic and kinetic indices are shown in Table 2. The Pearson correlation results indicate that during the First Hit process, there is a significant positive correlation between the Peak foot horizontal plane linear velocity of the non-dominant leg (*r* = 0.652, *p* < 0.05), Peak hip flexion angular velocity of the dominant leg (*r* = 0.736, *p* < 0.01), and Peak knee abduction angle of the dominant leg (*r* = 0.683, *p* < 0.05) with the effective score. There is also a significant negative correlation between the Peak ankle flexion angular velocity of the non-dominant leg (*r* = −0.637, *p* < 0.05), and Peak foot sagittal plane linear velocity of the non-dominant leg (*r* = −0.668, *p* < 0.05), and Peak hip abduction angle of the non-dominant leg (r = −0.767, *p* < 0.01), and Peak hip flexion angle of the dominant leg (*r* = −0.652, *p* < 0.05) with the effective score. In the second kick process, there were significant positive correlations between the Peak ankle pronation angular velocity of the dominant leg (*r* = 0.650, *p* < 0.05), peak foot coronal plane linear velocity of the dominant leg (r = 0.660, *p* < 0.05), peak hip adduction angle velocity of the dominant leg (*r* = 0.609, *p* < 0.05), and peak ankle internal rotation moment of the non-dominant leg (*r* = 0.587, *p* < 0.05) with effective scores. There was a significant negative correlation between the peak hip flexion angle of the dominant leg (*r* = −0.705, *p* < 0.01) and effective scores.

**TABLE 2:** Lower limb kinematic and kinetic index correlation results.

Course of action	Critical feature	Pearson correlation analysis		Stepwise regression analysis	
		R	*p*	*R* ^2^	DW
First Hit	Peak ankle flexion angular velocity of the non-dominant leg *	−0.637	0.026	0.406 (*n* = 12)	1.894
	Peak foot sagittal plane linear velocity of the non-dominant leg *	−0.668	0.018	0.446 (*n* = 12)	1.973
	Peak foot horizontal plane linear velocity of the non-dominant leg *	0.652	0.022	0.426 (*n* = 12)	1.524
	Peak hip abduction angle of the non-dominant leg **	−0.767	0.004	0.588 (*n* = 12)	1.796
	Peak hip flexion angle of the dominant leg *	−0.652	0.022	0.426 (*n* = 12)	1.331
	Peak hip flexion angular velocity of the dominant leg **	0.736	0.006	0.496 (n = 12)	1.421
	Peak knee abduction angle of the dominant leg *	0.683	0.014	0.466 (n = 12)	1.486
Second Hit	Peak ankle pronation angular velocity of the dominant leg *	0.650	0.022	0.422 (*n* = 12)	1.535
	Peak foot coronal plane linear velocity of the dominant leg *	0.660	0.020	0.435 (*n* = 12)	2.362
	Peak hip flexion angle of the dominant leg *	−0.705	0.010	0.498 (*n* = 12)	1.717
	Peak hip adduction angle velocity of the dominant leg *	0.609	0.036	0.371 (*n* = 12)	1.330
	Peak ankle internal rotation moment of the non-dominant leg *	0.587	0.045	0.344 (*n* = 12)	1.727

Notes: According to the Durbin-Watson test table, when *k* = 1 and *n* = 12, the lower critical value (dL) is 0.67 and the upper critical value (dU) is 1.023, If the Durbin-Watson (DW) statistic is greater than 1.023, it is deemed that the residuals meet the condition. A statistically significant difference was defined as *p* < 0.05. * indicates *p* < 0.05, ** indicates *p* < 0.01. Correlation strength (absolute value): 0.8–1.0 extremely strong correlation; 0.6–0.8 strong correlation; 0.4–0.6 moderate correlation; 0.2–0.4 weak correlation; 0.0–0.2 very weak or no correlation.

The results of the stepwise regression analysis (as shown in Table 3) indicate that during the first strike, the following variables have a statistically significant impact on the effective score: Peak ankle flexion angular velocity of the non-dominant leg (*R* ^*2*^ = 0.406, DW > 1.023), Peak foot sagittal plane linear velocity of the non-dominant leg (*R* ^*2*^ = 0.446, DW > 1.023), Peak foot horizontal plane linear velocity of the non-dominant leg (*R* ^*2*^ = 0.426, DW > 1.023), Peak hip abduction angle of the non-dominant leg (*R* ^*2*^ = 0.588, DW > 1.023), Peak hip flexion angle of the dominant leg (*R* ^*2*^ = 0.426, DW > 1.023), Peak hip flexion angular velocity of the dominant leg (*R* ^*2*^ = 0.496, DW > 1.023), and Peak knee abduction angle of the dominant leg (*R* ^*2*^ = 0.466, DW > 1.023). During the second strike, the following variables have a statistically significant impact on the effective score: Peak ankle pronation angular velocity of the dominant leg (*R* ^*2*^ = 0.422, DW > 1.023), Peak foot coronal plane linear velocity of the dominant leg (*R* ^*2*^ = 0.435, DW > 1.023), Peak hip flexion angle of the dominant leg (*R* ^*2*^ = 0.498, DW > 1.023), Peak hip adduction angle velocity of the dominant leg (*R* ^*2*^ = 0.371, DW > 1.023), and Peak ankle internal rotation moment of the non-dominant leg (*R* ^*2*^ = 0.344, DW > 1.023).

**TABLE 3:** Summary of stepwise regression analysis model for the relationship between lower limb kinematic, kinetic indices, and effective scores.

Model	R	*R* ^2^	Adjusted *R* ^2^	Durbin-Watson
1	0.767^a^	0.588	0.546	
2	0.887^b^	0.786	0.739	
3	0.950^c^	0.902	0.865	
4	0.943^d^	0.890	0.865	2.292

Notes:

^a^ Predictor Variable: Peak hip abduction angle of the non-dominant leg.

^b^ Predictor Variable: Peak hip abduction angle of the non-dominant leg, Peak ankle pronation angular velocity of the dominant leg.

^c^ Predictor Variable: Peak hip abduction angle of the non-dominant leg, Peak ankle pronation angular velocity of the dominant leg, Peak foot horizontal plane linear velocity of the non-dominant leg.

^d^ Predictor Variable: Peak ankle pronation angular velocity of the dominant leg, Peak foot horizontal plane linear velocity of the non-dominant leg.

^e^Implicit Variable: Effective Score.

### Stepwise regression results of lower limb kinematic and kinetic indicators with effective score

We further established a regression model based on the results of relevancy analysis. In this stepwise regression analysis, a total of 4 iterations of input or removal were performed. For the models created based on the 4 iterations, Models 3 and 4 achieved an adjusted R-squared of 0.86 or higher, indicating high goodness-of-fit of these models.

Based on the results in Table 4, it can be observed that all four equations derived from the stepwise regression analysis are statistically significant. However, in terms of model fit, equation 3 and (4) show better goodness-of-fit. Additionally, examining the coefficient test results reveals that the variable “Peak foot horizontal plane linear velocity of the non-dominant leg” in equation 3 is not statistically significant (*p* > 0.01), suggesting a preference for utilizing equation 4. The stepwise regression equation can be formulated using the B values of unstandardized coefficients in the following manner:

$$Y=23.492+1.817X_{1}+2.531X_{2}$$

$X_{1}$ refers to the peak ankle pronation angular velocity of the dominant leg, while $X_{2}$ represents the peak foot horizontal plane linear velocity of the non-dominant leg.

**TABLE 4:** Fitting status of the stepwise regression analysis model for the relationship between lower limb kinematic, kinetic indices, and effective scores.

Model		Unstandardized coefficient		Standardized coefficient	T	Sig
		B	standard error	Beta		
1	Constants	28.720	5.488		5.233	0.000
	Peak hip abduction angle of the non-dominant leg	−0.472	0.125	−0.767	−3.775	0.004
2	Constants	26.388	4.243		6.220	0.000
	Peak hip abduction angle of the non-dominant leg	−0.389	0.099	−0.631	−3.915	0.004
	Peak ankle pronation angular velocity of the dominant leg	1.241	0.429	0.466	2.891	0.018
3	Constants	22.755	3.270		6.959	0.000
	Peak hip abduction angle of the non-dominant leg	−0.114	0.114	−0.185	−.999	0.347
	Peak ankle pronation angular velocity of the dominant leg	1.656	0.337	0.621	4.918	0.001
	Peak foot horizontal plane linear velocity of the non-dominant leg	2.018	0.657	0.546	3.071	0.015
4	Constants	23.492	3.185		7.375	0.000
	Peak ankle pronation angular velocity of the dominant leg	1.817	0.295	0.682	6.152	0.000
	Peak foot horizontal plane linear velocity of the non-dominant leg	2.531	0.410	0.685	6.175	0.000

## Discussion and implications

This study systematically identifies the key features that contribute to the effective execution of the DRK technique, which leads to scoring points. These unique findings will provide coaches with evidence to systematically refine the high-difficulty techniques utilized by taekwondo athletes in competition, aiming to improve their success rate in landing kicks and ultimately enhance their winning potential in taekwondo competitions.

Among the 104 indicators we have identified, a total of 12 indicators (11.5%) demonstrate significant correlations with the effective scores. These significant interactions suggest key features that exhibit a strong and highly correlated relationship with the effective scores, providing potential guidance on improving the double roundhouse kick technique. Based on the characteristics of these 12 variables (4 variables related to joint motion velocity and acceleration, 4 variables related to joint angles, 3 variables related to terminal segment velocity, and 1 variable related to joint torque), they can be defined as 2 key features: (1) joint motion and (2) stable support. The biomechanical variables included in these two key features are as follows, Joint motion: Refers to variables related to terminal segment velocity, joint motion velocity and acceleration. Stable support: Refers to variables related to joint angles and joint torque. The following will discuss the critical features and how they are believed to contribute to the successful performance of the DRK technique.

### Joint motion

According to the research results on relevance, among the 12 key indicators, there are 4 indicators related to joint flexion and extension. Therefore, we categorize these four indicators as follows: joint flexion and extension. This study demonstrated a significant relationship between the angular velocity of ankle joint extension during kicking, the hip joint extension angle and angular velocity of the supporting leg, and effective scoring in the kicking process. Higher angular velocity of ankle joint extension during kicking and greater hip joint extension angle of the supporting leg were found to be detrimental to effective scoring, while higher hip joint extension angular velocity of the supporting leg was found to be beneficial for triggering a score. Gavagan and Sayers. (2017) discovered that in a kicking motion, the generation of kinetic energy by the supporting leg resulted in hip rotation and forward tilt, thereby improving kicking performance. This finding contradicts previous research that found an increased hip joint tilt angle of the supporting leg when athletes attempted to expand their kicking range without losing speed (Kim et al., 2010; Cortes et al., 2011). To execute DRK, the supporting leg must initiate before the striking leg makes contact with the ball and utilize hip joint flexion torque to drive knee extension, thereby enhancing the kicking action of the striking leg. This requires hip joint flexion to provide the necessary potential energy for the kick, further supporting the conclusions of this study. The ankle joint of the striking leg has a significant influence on the final triggering of a score. The flying side kick is a whipping motion where the sequence of joint actions occurs from proximal to distal segments (Estevan et al., 2015). The whipping motion drives the distal segment by generating torque at the proximal segment (Fong and Tsang, 2012), and skilled athletes primarily control the ankle joint by controlling the pelvis, knee, and ankle joints. A higher angular velocity of the supporting leg’s knee joint extension can provide greater potential energy for the hip joint of the striking leg, increasing the distal velocity required for scoring. Huang et al. (2022) also share this view, stating that skilled athletes can achieve a high impact index for early rotation kicks by increasing the linear velocity of the proximal segments (femur and tibia) at the same level of ankle joint velocity.

This text describes some biomechanical research findings on DRK that may affect the effectiveness of scoring. When the first strike is performed, the closer the body is to the target, the shorter the distance for the second strike relative to the first strike, making it more difficult to score successfully. The ability to score successfully depends on whether the knee joint of the attacking leg can fully extend when the striking distance is reduced. At the same time, the change in striking distance places high demands on the athlete’s ability to adapt to the variation. Kim et al. (2010) found that top athletes are able to execute kicks with comparable power at shorter distances. Chang et al. (2021) observed in their study on side kicks that the supporting leg of the athlete maintains contact with the ground throughout the striking process, and this foot-ground connection becomes more solid after impact. This is because after completing the body rotation action, the relative movement between the athlete and the ground is greatly reduced, increasing the effective mass of the attacking leg and transferring more momentum to the attack, making the DRK more powerful. This study suggests that the first strike in DRK primarily provides potential energy for the second strike and helps lift the body off the ground. The second strike is performed when the body is off the ground, and the landing of the supporting leg is mainly to provide stable support for the body.

### Stable support

This study found that there is a significant positive correlation between the inversion angular velocity of the ankle joint, the abduction angle of the knee joint, the abduction angular velocity of the hip joint, and the effective score value when acting as the supporting leg. However, there was no significant correlation between the inversion and eversion moments of the ankle joint and the effective score value. This result may negate our hypothesis. The reason could be that at the moment of impact with the target by the attacking leg, it experiences an impact force of approximately 3482 N (Falco et al., 2009). If the ankle joint generates a large flexion/extension moment, it cannot effectively resist the backward movement of the body, leading to a significant shift in the center of gravity and affecting the athlete’s body balance (Flanagan and Salem, 2008; Falco et al., 2009). On the other hand, the supporting leg can prevent the body from moving backward after the impact of the attacking leg. Before the impact of the attacking leg on the target, the hip joint of the supporting leg rotates rapidly and drives the rotation of the lower limb. This helps to avoid the supporting leg from getting “stuck” on the ground and enables the body to rotate and maintain a stable striking posture (Mihata et al., 2006; Brophy et al., 2010; Moreira et al., 2017). Restricting the inversion and eversion movements of the ankle joint of the supporting leg during the strike can “lock” the ankle joint, making the kicking action more effective. In Taekwondo competitions, generating high terminal impact velocity is crucial, which requires coordination between the lower body muscles and bones (Gulledge and Dapena, 2008).

We found that there was no significant correlation between vertical ground reaction force and effective score value, which contradicts our hypothesis. Based on these findings, to increase distal speed and reduce the impact effect, it is necessary to increase the angular velocity of the hip joint in the vertical axis and decrease the angular velocity in the sagittal axis, preventing the flexion moment of the hip joint from being transmitted from the proximal to the distal end. The study results indicate that a more open or closed sagittal, coronal, or vertical axis on the hip joint of the attacking leg is unfavorable for scoring, and athletes should ensure hip joint locking to enhance stability during impact. Therefore, stability of the attacking leg during impact is crucial for triggering a score. In Taekwondo competitions, for a kick to be effective, athletes must strike the opponent with maximum speed and power (Falco et al., 2013; Estevan et al., 2015). The contact surface area between the foot and electronic body protector (PSS) directly affects the final triggering score, as demonstrated by Machado et al. (2010); the vertical component of the foot mannequin contact is influenced by the ankle joint’s inward and outward movement at the moment of impact, resulting in a reduced contact area between the foot and mannequin, thus affecting the score. This study shows a significant positive correlation between knee inversion/eversion angle and effective score value. With distal speed reaching the threshold for an effective score, the vertical contact area between the foot and target is the most important factor influencing the score. As the ankle joint velocity has already reached its maximum value before hitting the target, athletes must adjust the foot angle of the attacking leg before impact to ensure sufficient contact with the target. More study into the biomechanical mechanism of the ground response force of the DRK is required since this may explain why there was no significant difference between the ground reaction force and the effective score value at the time of the two blows.

In summary, the key factors contributing to effective scoring in electronic armor are joint flexion/extension and stable support. Taekwondo coaches and athletes can develop targeted training plans focusing on the muscle strength of the lower extremity joints to improve the ratio of effective scoring in competitive matches. Due to the limitations of this study, it only focuses on kinematic and kinetic indicators such as joint angles, angular velocities, torques, and ground reaction forces. Future research is needed to confirm the effect of lower extremity muscle force on effective scoring. We plan to utilize the Opensim musculoskeletal simulation model to simulate the lower limb muscle force during the execution of a roundhouse kick, and integrate kinematic and kinetic indicators to identify the optimal muscle coordination pattern for the lower limb during this action, thereby confirming the impact of lower limb muscle force on effective scoring.

## Conclusion

In the process of striking, explosive power, body posture, adequate terminal velocity, and body rotation are key features that directly influence effective scoring with electronic protective gear. Among them, the peak ankle pronation angular velocity of the dominant leg and the peak foot horizontal plane linear velocity of the non-dominant leg make significant contributions to effective scoring with electronic protective gear. The derived stepwise regression equation is: Y = 23.492 + 1.817 peak ankle pronation angular velocity of the dominant leg +2.531 peak foot horizontal plane linear velocity of the non-dominant leg.

## Data Availability

The original contributions presented in the study are included in the article/Supplementary Material, further inquiries can be directed to the corresponding author.
